# Mineralizing Filamentous Bacteria from the Prony Bay Hydrothermal Field Give New Insights into the Functioning of Serpentinization-Based Subseafloor Ecosystems

**DOI:** 10.3389/fmicb.2017.00057

**Published:** 2017-01-31

**Authors:** Céline Pisapia, Emmanuelle Gérard, Martine Gérard, Léna Lecourt, Susan Q. Lang, Bernard Pelletier, Claude E. Payri, Christophe Monnin, Linda Guentas, Anne Postec, Marianne Quéméneur, Gaël Erauso, Bénédicte Ménez

**Affiliations:** ^1^Geomicrobiology Group, Institut de Physique du Globe de Paris, Sorbonne Paris Cité, Université Paris Diderot, Centre National de la Recherche ScientifiqueParis, France; ^2^DISCO beamline, Synchrotron SOLEILSaint Aubin, France; ^3^Institut de Minéralogie, de Physique des Matériaux et de Cosmochimie, Institut de Recherche pour le Développement, Université Pierre et Marie CurieParis, France; ^4^Department of Earth and Ocean Sciences, School of the Earth, Ocean and Environment, University of South Carolina, ColumbiaSC, USA; ^5^GIS Grand Observatoire de l’environnement et de la biodiversité terrestre et marine dans le Pacifique Sud, Centre IRD de NouméaNouméa, New Caledonia; ^6^UR227 COREUS, Centre IRD de NouméaNouméa, New Caledonia; ^7^Géosciences Environnement Toulouse, Université Paul Sabatier, Centre National de la Recherche Scientifique, Institut de Recherche pour le DéveloppementToulouse, France; ^8^Laboratoire Matériaux Polymères Interfaces Environnement Marin EA 4323, Université de ToulonLa Garde, France; ^9^Mediterranean Institute of Oceanography, Centre IRD de NouméaNouméa, New Caledonia; ^10^Laboratoire Insulaire du Vivant et de l’Environnement, Université de la Nouvelle-CalédonieNouméa, New Caledonia; ^11^Aix Marseille Université, Centre National de la Recherche Scientifique – Institut National des Sciences de L’Univers, Université de Toulon, Institut de Recherche pour le Développement, Mediterranean Institute of OceanographyMarseille, France

**Keywords:** serpentinization, alkaline hydrothermalism, deep life, *Firmicutes*, *Acetothermia*, *Omnitrophica*, organic carbon

## Abstract

Despite their potential importance as analogs of primitive microbial metabolisms, the knowledge of the structure and functioning of the deep ecosystems associated with serpentinizing environments is hampered by the lack of accessibility to relevant systems. These hyperalkaline environments are depleted in dissolved inorganic carbon (DIC), making the carbon sources and assimilation pathways in the associated ecosystems highly enigmatic. The Prony Bay Hydrothermal Field (PHF) is an active serpentinization site where, similar to Lost City (Mid-Atlantic Ridge), high-pH fluids rich in H_2_ and CH_4_ are discharged from carbonate chimneys at the seafloor, but in a shallower lagoonal environment. This study aimed to characterize the subsurface microbial ecology of this environment by focusing on the earliest stages of chimney construction, dominated by the discharge of hydrothermal fluids of subseafloor origin. By jointly examining the mineralogy and the microbial diversity of the conduits of juvenile edifices at the micrometric scale, we find a central role of uncultivated bacteria belonging to the *Firmicutes* in the ecology of the PHF. These bacteria, along with members of the phyla *Acetothermia* and *Omnitrophica*, are identified as the first chimneys inhabitants before archaeal *Methanosarcinales*. They are involved in the construction and early consolidation of the carbonate structures *via* organomineralization processes. Their predominance in the most juvenile and nascent hydrothermal chimneys, and their affiliation with environmental subsurface microorganisms, indicate that they are likely discharged with hydrothermal fluids from the subseafloor. They may thus be representative of endolithic serpentinization-based ecosystems, in an environment where DIC is limited. In contrast, heterotrophic and fermentative microorganisms may consume organic compounds from the abiotic by-products of serpentinization processes and/or from life in the deeper subsurface. We thus propose that the *Firmicutes* identified at PHF may have a versatile metabolism with the capability to use diverse organic compounds from biological or abiotic origin. From that perspective, this study sheds new light on the structure of deep microbial communities living at the energetic edge in serpentinites and may provide an alternative model of the earliest metabolisms.

## Introduction

Active microbial life requires thermodynamic disequilibria and geochemical gradients, which are determined by the availability of electron donors and acceptors (Branscomb and Russell, 2013; Colwell and D’Hondt, 2013). In this regard, dynamic geological systems such as hydrothermal vents and serpentinizing environments may sustain microbial activity, making the deep ocean basement a potentially large microbial habitat. In these environments, Subsurface Lithoautotrophic Microbial Ecosystems (i.e., SLIMEs) may exist and persist independently from photosynthesis by using energy and carbon solely from geological sources (Nealson et al., 2005). Thus they are relevant to document the quest for the earliest type of biomass-generating metabolism. If one can assume a continuity of microbial metabolisms from their inception to the present day, autotrophic archaeal methanogenesis along with bacterial homoacetogenesis constitute likely potential ancestral metabolisms in the alkaline hydrothermal theory for the origin of life (Nitschke and Russell, 2013). They both implement metabolic pathways that could have derived from geochemical forerunners spontaneously occurring during the hydration of oceanic mantle-derived rocks (Sleep et al., 2004; Martin and Russell, 2007). Indeed, as soon as water circulated through the ultramafic komatiitic crust of the early Earth (Arndt and Nisbet, 2012), serpentinization, *via* the oxidation of ferrous iron bearing minerals, produced abundant H_2_ that could have, in turn, reduced inorganic carbon in an overall exergonic reaction (e.g., CO_2_ reduced to methane). This abiotic reaction shares enough chemical and catalytic similarities with the reductive acetyl-coenzymeA (Co-A) pathway used by methanogens and homoacetogens to potentially be at the origin of the first energy-harnessing biochemical pathways that underpin microbial growth (Shock and Schulte, 1998; Amend and McCollom, 2009). Advances in studies of subsurface microbial life as analogs of primitive ecosystems strongly depend on the accessibility of subseafloor basement habitats. This can be indirectly and partly achieved through the sampling and characterization of the hydrothermal fluids that discharge at the ocean floor or in continental ophiolitic sites, as open windows to the subsurface (Cowen, 2004; Brazelton et al., 2012).

From that perspective, the discovery in 2000 of the off-axis serpentinization-related Lost City Hydrothermal Field (LCHF) near the Mid-Atlantic Ridge (30°07′N; Kelley et al., 2001) and the established presence of abiotic methane in its highly reduced eﬄuents (Proskurowski et al., 2008) went far in changing our vision of how life has emerged on Earth. In this hyperalkaline environment (pH 9–10.8), moderate-temperature (40–90°C) fluids discharge at the seafloor and lead to the formation of carbonate chimneys. The continuously supplied serpentinization by-products, including hydrogen, short chain hydrocarbons, and formate, sustain microbial ecosystems that produce, among other organic compounds, acetate (Brazelton et al., 2006; Lang et al., 2010; Schrenk et al., 2013). These chimneys were shown to grow from porous and fragile structures made of interlacing networks of aragonite and brucite minerals to more consolidated edifices *via* calcite precipitation (Ludwig et al., 2006). Brucite-dominated hydrothermal chimneys were also recently described in the serpentinizing environment of the southern Mariana forearc (Okumura et al., 2016). Although lacking precise descriptions, traces of filamentous microorganisms were noticed in both cases in these carbonated structures, raising the question of the impact of microorganisms on chimney construction processes. At LCHF, in the highest pH and anoxic conduits of actively venting chimneys, dense biofilms were dominated by a single uncultured phylotype of methane-metabolizing archaea referred to as Lost City *Methanosarcinales* (LCMS; Schrenk et al., 2004; Brazelton et al., 2006). A single kind or a syntrophic assemblage of organisms belonging to LCMS were shown to be involved in both methane production and oxidation by utilizing either H_2_ or abiotic methane, both of which are present at high concentrations at LCHF (1–15 and 1–2 mM, respectively; Brazelton et al., 2011; Schrenk et al., 2013). The methanogenic/methanotrophic LCMS were then presented as the primary producers in the ecology of these reduced hyperalkaline environments, hence supporting the whole microbial ecosystem. *Methanosarcinales* were also described in other environments related to active serpentinization zones including alkaline subterrestrial environments (Schrenk et al., 2004; Kelley et al., 2005; Brazelton et al., 2012; Suzuki et al., 2013; Tiago and Verissimo, 2013). They may then constitute the base of a H_2_-driven SLIME. The variable physiological activity of the monophyletic LCMS together with their functional gene diversity and morphological differentiation raise questions about the possibility for more diverse metabolisms and carbon assimilation pathways in this order (Brazelton et al., 2011).

If the discovery of the LCHF was surprising because of its uniqueness, serpentinite-hosted hydrothermal circulation is nonetheless widespread along mid-ocean ridges and in ophiolitic massifs. Hence, similar environments are likely not scarce on the modern Earth and may also have existed since the Archean (Schrenk et al., 2013). The closest currently known analog of the LCHF is the Prony Bay Hydrothermal Field (PHF, New Caledonia) known for its thermal springs (Rivière des Kaoris and Bain des Japonais) and the famous Prony spire (Roc Aiguille de la baie de Prony; Magnier, 1979; Launay and Fontes, 1985) in the northernmost part of the bay. More recently numerous active structures (about 50 of them having significant metric size) were found across the entire bay (Pelletier and Chevillon, 2006; Pelletier and Payri, 2010). PHF develops at shallow water depth as the result of the discharge in a marine lagoon of high-pH waters of meteoric origin that have percolated through the ophiolitic ultramafic substratum. Due to its accessibility by scuba diving, the PHF offers the possibility of long-term monitoring, intensive sampling, and spatial comparisons between vents (Supplementary Figure S1; Pelletier et al., 2011). The geochemistry and microbiology of the PHF were recently found to have strong similarities with the LCHF in terms of pH, H_2_, and CH_4_ emissions and microbial community structure (Monnin et al., 2014; Quéméneur et al., 2014; Postec et al., 2015) despite fluids having lower temperatures (<40°C). The archaeal sequences retrieved from the PHF were dominated by two phylotypes of the *Methanosarcinales* order, namely the LCMS and an additional representative first identified in The Cedars subterrestrial serpentinization-driven ecosystem (USA; Suzuki et al., 2013).

Similar to LCHF, the PHF ecosystem represents an open window to investigate geochemical and biological processes occurring at depth in a serpentinization-based SLIME, with implications for the early evolution of life. A dense and diverse microbial community develops in the chimneys where hydrothermal fluids mix with seawater, creating strong chemical and redox gradients. Yet, it remains challenging to identify, among the large diversity of species, those organisms that represent true SLIME members. The main purpose of this study was to investigate the subsurface microbial ecology of the PHF serpentinizing environment and the influence of organomineralization/biomineralization processes on chimney construction. We focus on the most juvenile hydrothermal chimneys, in which discharged fluids are dominated by pristine hydrothermal fluids of subsurface origin. By using Scanning Electron Microscopy (SEM), laser microdissection combined with X-Ray Diffraction (XRD), Confocal Laser Scanning Microscopy (CLSM) coupled with Fluorescence *In Situ* Hybridization (FISH) experiments and phylogenetic analyses, we show that investigations at the micrometric scale allow us to better track microbial representatives of subsurface serpentinizing environments. We discovered that the populations associated with the most pristine hydrothermal fluids and involved in the early stages of chimney construction are not archaeal *Methanosarcinales.* Instead, they are filamentous bacteria whose study may provide new insights into the functioning of subsurface microbial ecosystems at the energetic edge in serpentinizing environments.

## Materials and Methods

### Samples Collection and Description

**FIGURE 1:**
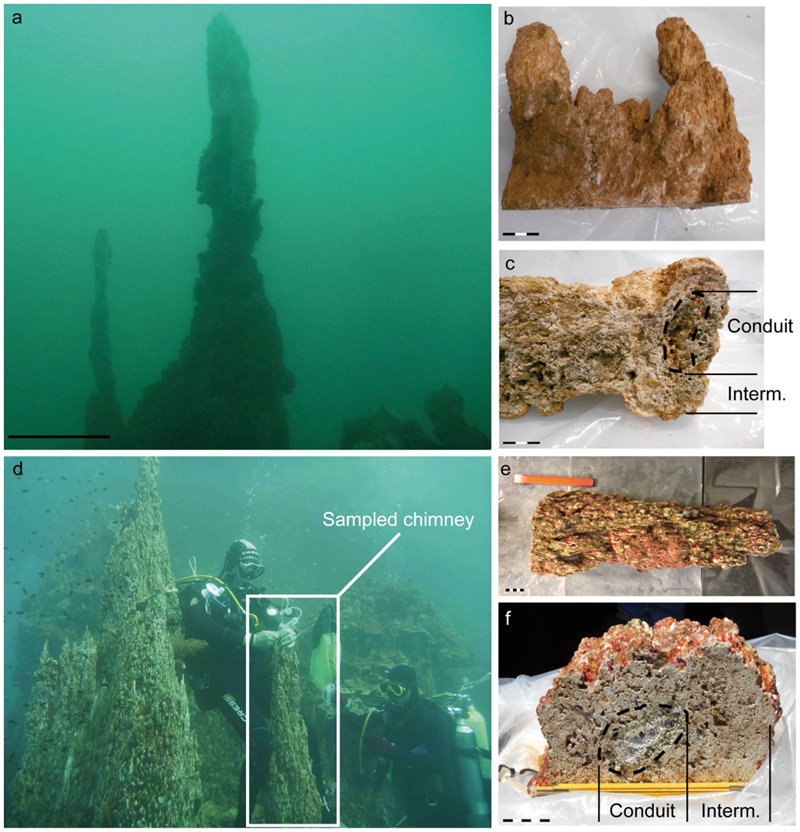
**Photographs of the hydrothermal edifices from the Prony Bay (New Caledonia) characterized in the present study and sampled by scuba divers in 2011: ST09 (a–c)**, a quite young and active carbonated vent hosted at 50 mbsl on serpentinites, and ST07 **(d–f)** also called Roc Aiguille of Prony as the more massive carbonated chimneys of the lagoon, located at 25 mbsl. **(a,d)** are dive photographs of sites ST09 and ST07, respectively (modified from Quéméneur et al., 2014 and Postec et al., 2015). **(b)** (ST09) and **(e)** (ST07) are detailed photographs of the sampled chimneys, both actively discharging fluids, and **(c)** (ST09) and **(f)** (ST07) are transversal cuts with the localization of the active hydrothermal conduits. The venting structures were commonly organized in three parts. First, the inner conduit of a few centimeters in diameter corresponded to the location of the circulation of the reduced and alkaline fluids, deriving from serpentinization processes at depth. Secondly, the intermediate part (white to green color) constituted the main walls of the chimneys. Finally, the external part of the chimneys in direct contact with seawater were often colonized by macrofauna and corals, but not always, depending on the age of the chimney **(a)**: scale bar = 1 m; **(b,c,e,f)**: black scale bar = 1 cm.

Hydrothermal chimneys studied and reported herein were recovered in 2011 during the multidisciplinary HYDROPRONY cruise (*R/V Alis*; Pelletier et al., 2011) in the Prony Bay (**Figure 1**). Sample sites were distributed on the seafloor at depths between 15 and 50 meters below sea level (mbsl) except for two sites located in the intertidal zone (Bain des Japonais and Rivière des Kaoris). The sites lie on serpentinized peridotites, gabbros, and dunites that form the main geological substratum of South New Caledonia (Supplementary Figure S1). The underwater structures, where high-pH hydrothermal fluids discharge and mix with seawater, were mainly made of brucite along with carbonates (Pisapia et al., 2013). They were organized as massive consolidated concretions that may take the form of needles up to 35 m high with, if active, small centimetric whitish and unconsolidated nascent protochimneys on their top (**Figure 1**; Supplementary Figure S2).

Twenty-five active and fossil vents (from eight different sites spanning the Prony Bay) were sampled for molecular ecology, geochemistry, and mineralogy during the HYDROPRONY expedition. We focus here on two types of active chimneys sampled at ST07 and ST09 sites, a few kilometers apart in the bay (**Figure 1**; Supplementary Figure S1). They are representative of two distinct development stages of the venting edifices. The youngest chimney came from ST09, a 50 m wide and 3 m high dome in the center of a 150 m wide depression at 50 mbsl, overlain by 3–4 m high chimneys with smaller active vents. The external part of the actively venting and poorly consolidated sampled chimney was not colonized by marine macrofauna, evidence that it is a young structure (**Figures 1b,c**). Additionally, except at the coastal sites (Bain des Japonais and Rivière des Kaoris), the fluid venting through the brucite-dominated chimney of ST09 appeared to be the most pristine among all the submerged sites, hence the most representative of the serpentinization-derived hydrothermal fluid (Monnin et al., 2014). Site ST07, the Roc Aiguille of Prony Bay, is a shallower and more massive structure with a main pinnacle ∼38 m high and culminating up to 2 mbsl. This is the tallest structure in the bay and probably the oldest. The sampled chimney was covered by a pronounced coral crust, attesting to a long residence time at the seafloor (**Figures 1e,f**). Chimney samples from sites ST08, ST11, ST12 and Bain des Japonais (Supplementary Figures S1 and S2) were also examined to assess the representativeness of our microscale observations. An overview of the studied sites and of the analyses performed on each is available in Supplementary Table S1. Full site descriptions along with vent fluid geochemistry and associated molecular ecology of some of the hydrothermal chimneys at PHF can be found in Pelletier et al. (2011), Monnin et al. (2014), Quéméneur et al. (2014) and Postec et al. (2015). Elemental compositions and temperatures of the fluids collected at the main selected sites are reported in Supplementary Table S2 where they are compared to the LCHF and The Cedars (Kelley et al., 2001; Suzuki et al., 2013).

Samples dedicated to SEM microimaging were extracted or cut aseptically in the field and immediately stored at 4°C. Samples for molecular ecology were collected on board using sterilized pliers immediately after the scuba dives and each sample was divided into several fractions. The fractions dedicated to FISH experiments were fixed with 2% formaldehyde in phosphate-buffered saline solution (PBS; Gérard et al., 2013) and stored in 50% ethanol/PBS at -20°C until use (see details in Postec et al., 2015). Other fractions of the same samples were immersed in 50% ethanol and kept at -20°C for further molecular characterizations or CLSM observations.

### Scanning Electron Microscopy

Scanning Electron Microscopy observations of freshly cut chimney fragments were performed at the Service Commun de Microscopie Electronique à Balayage (UPMC, Paris, France) using a Zeiss SUPRA 55 VP Field Emission Scanning Electron Microscope. Samples were Au-coated and no organic resin was employed. Images were collected using secondary electron detectors (Everhart-Thornley for high voltage mode, VPSE for variable pressure mode and InLens for low voltage mode) and a backscattered electron detector (AsB). Accelerating voltage ranged from 7 to 15 kV at variable pressures and high current (up to 1 nA) or was fixed at 3 kV under high vacuum and low current (down to 10 pA). Elemental microanalyses were also performed using an Energy Dispersive X-ray (EDX) spectrometer (PGT Sahara).

### Laser Microdissection and X-Ray Diffraction

In order to reliably determine the nature of the minerals that encrust microbial filaments (see Section “Organomineralization Processes Drive the Early Stage of Chimney Construction at the PHF”), we extracted individual mineralized biofilaments by laser microdissection using a Zeiss PALM MicroBeam apparatus. Filaments were microdissected from the most juvenile ST09 chimney samples and were recovered in sterile Eppendorf^TM^ tubes. Individual mineralized filaments were stuck on a borosilicate glass rod and were analyzed by XRD phi-scan at the IMPMC (UPMC, Paris, France). Diffractograms were obtained, after a 10 h acquisition time (step of 0.039° 2𝜃), with an Agilent XCalibur S diffractometer equipped with a four-circle goniometer, a Sapphire 3 CCD detector and a monochromatic Mo X-ray source (0.71073 Å).

### Epifluorescence Microscopy, Confocal Laser Scanning Microscopy, and Fluorescent *In situ* Hybridization Experiments

Epifluorescence microscopy was carried out on an Olympus BX51 microscope equipped with an Olympus UPlanApo 40×/1.00 Oil Iris objective and a U-MNIB3 filter after Syto^®^9 staining of the samples (Invitrogen). CLSM was carried out using an Olympus FluoView FV1000 confocal microscope with an oil immersion objective Olympus UPSLAPO 60xO (Gérard et al., 2013). Fluorescence image stacks were obtained with concomitant excitation at wavelengths of 405, 488, and 543 nm by collecting the emitted fluorescence between 425–475, 500–530, and 560–660 nm, respectively. Three-dimensional images were acquired, visualized, and processed using the F10-ASW FLUOVIEW software (Olympus). Microbial cells were localized and imaged thanks to non-specific DNA staining using green-fluorescent Syto^®^9 dye (excitation at 488 nm and emission detection range 500–530 nm). A set of at least four stained subsamples coming from the same conduit were examined each time. The autofluorescence properties of the F_420_ factor involved in the energy metabolism of anaerobic methane-cycling archaea were also collected after excitation at 405 nm (detection range 420–480 nm). The associated fluorescence spectra (data not shown) were recorded following the protocol described in Postec et al. (2015). Complementary FISH experiments were conducted on mature chimneys samples in order to document the presence of *Methanosarcinales-*related cells. The oligonucleotide probes (Thermo Fisher Scientific Inc., Waltham, MA, USA) used were specific for *Euryarchaeota* (EURY498, 5′-CTTGCCCRGCCCTT-3′) and *Crenarchaeota* (CREN499, 5′-CCAGRCTTGCCCCCCGCT-3′; Burggraf et al., 1994). More details are available at probeBase (Loy et al., 2007). The hybridization experiments were conducted on multitest slides (10 wells) at 46°C for 2–4 h on formaldehyde-fixed chimney fragments from site ST07 using 5 ng⋅μL^-1^ of each probe in 0.9 M NaCl, 20 mM Tris-HCl pH 8, 0.01% SDS containing 35% (vol/vol) formamide. Samples were then washed for 15 min at 48°C in 0.9 M NaCl, 20 mM Tris pH 8.5 mM EDTA, 0.01% SDS. The slides were then soaked in cold water for a few seconds and air-dried. Finally, samples were stained with DAPI (excitation at 405 nm and detection range between 450 and 500 nm) at a concentration of 1 μg⋅mL^-1^ (Sigma) for 1 min, then washed for a few seconds in cold water, and left to dry. For CLSM examination, hybridized cells were covered by Citifluor AF3 (glycerol mounting solution from Emgrid).

### Filament Phylogenetic Identification

When bacterial filamentous microorganisms were abundantly detected using CLSM and epifluorescence microscopy (see section “Organomineralization Processes Drive the Early Stage of Chimney Construction at the PHF”), DNA extractions were performed with the PowerSoil^®^DNA Isolation Kit (Mo Bio, USA). For this purpose, a total volume of sample of about 1 cm^3^ was dissected from the filament-rich areas of the most juvenile ST09 chimney samples. The extracted sample was divided into two subsamples. The first one was stained with Syto^®^9 to ensure using epifluorescence microscopy the abundance of filamentous microorganisms. The second aliquot was kept unstained. DNA extractions were performed on both. Due to the small amount of DNA recovered with this procedure, DNA was also extracted from bulk rock bathed by the hydrothermal fluids in ST09 and ST11 chimney samples (hereafter referred to as bulk chimney samples). This provided a broader overview of the microbial population hosted in the active conduits of ST09 and ST11 juvenile chimneys. In both cases, bacterial 16S rRNA genes were amplified by PCR using (i) the bacterial specific primer 27F (5′-AGAGTTTGATCCTGGCTCAG-3′) with the prokaryote specific reverse primer 1492R (5′-GGTTACCTTGTTACGACTT-3′) and (ii) the bacterial specific primer 63F (5′-CAGGCCTAACACATGCAAGTC-3′) with the prokaryote specific reverse primer 1387R (5′-GGGCGGWGTGTACAAGGC-3′). All primers were provided by GATC (France). Five microliters of purified DNA were used in a reaction buffer volume of 25–30 μL containing 1.5 mM MgCl_2_, dNTPs (10 nmol each), 20 pmol of each primer and 1 U GoTaq polymerase (Promega, France). PCR reactions were performed under the following conditions: 35 cycles (denaturation at 94°C for 15 s, annealing at 55°C for 30 s, extension at 72°C for 2 min) preceded by 2 min denaturation at 94°C, and followed by 7 min extension at 72°C. Direct PCR amplifications were carried out with different primer combinations. For the dissected filament-rich samples, we performed in addition nested PCR. 1 μL of the products of the first PCR carried out with the most external primers (i.e., 27F and 1492R) was used in nested PCRs with primers 63F and 1387R and using the same conditions (35 cycles – denaturation at 94°C for 15 s, annealing at 55°C for 30 s, extension at 72°C for 2 min – preceded by 2 min denaturation at 94°C, and followed by 7 min extension at 72°C). The controls for all PCRs were negative, including the controls from the first PCR used as template for the nested PCR. Cloning was done using the Topo TA Cloning system (Invitrogen) following the instructions provided by the manufacturer. After plating, positive transformants were screened by PCR amplification of inserts using flanking vector primers and the PCR products were partially sequenced using either 1387R or 1492R. We first examined the 16S rRNA gene sequence of 41 clones from four different cloning experiments, two derived from the DNA extracted from the Syto^®^9-stained sample, which was examined with CLSM and two derived from the unstained second-half of the sample. We obtained a larger set of clones from DNA bulk extractions (88 and 87 for ST09 and ST11, respectively) and then examined these larger sets of sequences. PCR amplifications of archaeal 16S rRNA genes were also attempted from DNA extracted from ST09 and ST11 chimney samples. While the primers were the same as in Postec et al. (2015) where *Archaea* sequences were retrieved, they gave no results in this study. Following the protocol described in Gérard et al. (2009), all sample manipulations were done using material dedicated to samples with high contamination risks. PCR protocols were realized using a Biocap^TM^ RNA/DNA hood (Captair, Erlab, France) equipped with a HEPA filter. Chamber and micropipettes were UV-sterilized before use and aerosol resistant pipette tips were used to reduce external contamination.

The sequences reported in this paper have been deposited in the GenBank database. Sequences retrieved from the unstained and Syto^®^9-stained ST09 samples on which CLSM observations were carried out correspond to the accession no. KM207235 for HPst091-1-1 and KR911715, KR911716, and KR911717 for St09-1-17, St09-2-3, and ST09S-2, respectively. The sequence HPst091-1-1 was retrieved after direct PCRs (primers 63F and 1387R) and nested PCRs (primers 27F-1492R then 63F-1387R) from both Syto^®^9-stained and unstained samples, St09-1-17 and St09-2-3 only after nested PCR from the unstained sample, and ST09S-2 after direct PCR from the Syto^®^9-stained sample. Sequences obtained from bulk chimney samples have accession no. ranging from KX344725 to KX344743 and KX349203 for ST09 and from KX344744 to KX344775 for ST11.

### Phylogenetic Analyses

Taxonomic affiliations at the phylum level were first obtained by comparing several portions of partial 16S rRNA gene sequences with sequences of the GenBank database using BLAST (Basic Local Alignment Search Tool; Altschul et al., 1997). Representative clones of the dominant phyla were then fully sequenced and analyzed with the ARB software (Ludwig et al., 2004) by using the SILVA database SSU ref LSU 123 – full release (Pruesse et al., 2007; Quast et al., 2013; Yilmaz et al., 2014). The sequences were first aligned with the SINA online aligner (Pruesse et al., 2012) and then added in the ARB guide tree using the ARB parsimony tool. The phylogenetic tree was then constructed by adding to the aligned sequences, sequences of the closest cultivated bacteria and environmental clones in the RAxML (Randomized Axelerated Maximum Likelihood) program (Stamatakis et al., 2008) by using the GTRCAT substitution model. The bootstrap values were calculated from 1,000 replicates.

### Organic Acid Analysis

Organic acid concentrations were analyzed in duplicate using the method of Albert and Martens (1997). Error of individual measurements was ± 0.5 μM and corresponded to the larger of either the standard deviation between the two analyses, or the individual measurement error. Samples were analyzed for formate, acetate, propanoate, butyrate. Only formate and acetate were detected at concentrations greater than 0.5 μM.

## Results and Discussion

### Organomineralization Processes Drive the Early Stage of Chimney Construction at the PHF

Both mineralogical and microbiological investigations were conducted on the chimney internal conduits (**Figures 1c,f**; Supplementary Figure S2d) where the reduced and alkaline fluids, derived from serpentinization at depth, circulated. They hosted brown microbial mats on which our investigations focused. Similar to what has been described for the LCHF (Schrenk et al., 2004; Ludwig et al., 2006) the actively venting chimneys were highly porous. The interior of the chimneys was dominated by brucite mixed with Mg-carbonates and aragonite, with an increasing proportion of Ca-carbonates closer to the exterior of the chimney, which precipitated when seawater started to mix with the hyperalkaline hydrothermal fluids (Pisapia et al., 2013; Monnin et al., 2014).

**FIGURE 2:**
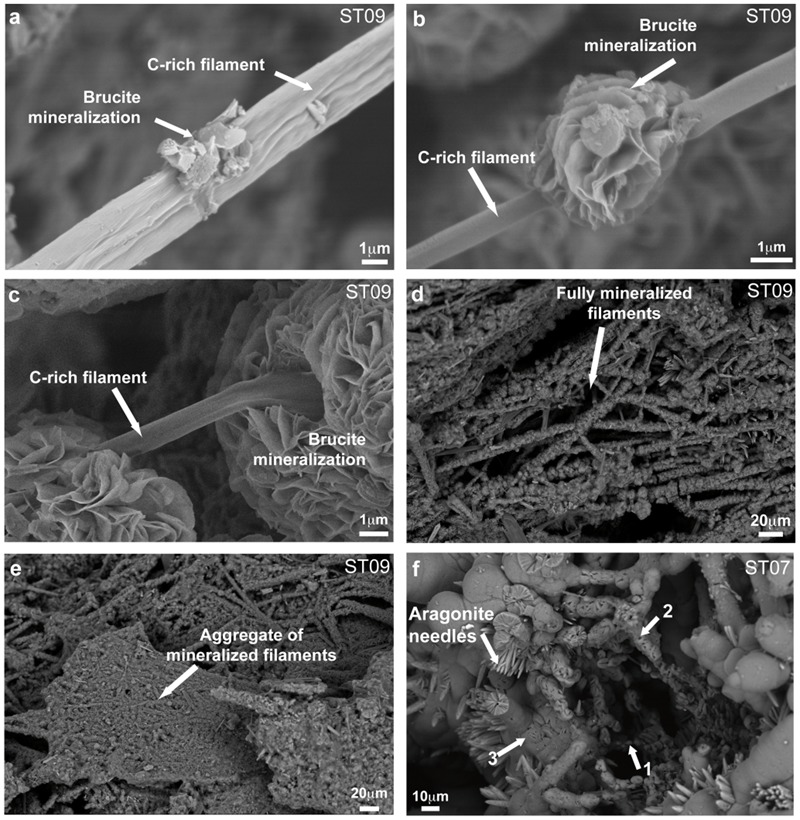
**Representative SEM images of the progressive mineralization of C-rich filaments in the active conduits of a juvenile chimney sampled at site ST09 (a–e)**, hence leading to a compact assemblage consolidating the nascent edifice. Those are preserved in more mature chimneys as the one sampled at site ST07 **(f)**. **(a)** Initial stage of mineralization with first precipitates being visible at the surface of a C-rich filament (collected at 7 kV in AsB mode). **(b–c)** Ongoing aggregation of brucite crystals along a filament [**(b)** at 7 kV, AsB mode and **(c)** at 3 kV, InLens mode]. **(d–e)** Fully-mineralized filaments forming a 3D porous structure that densified during early vent construction to form a compact chimney wall (15 kV, AsB mode). **(f)** Similar encrusted filaments preserved in the conduits of a mature chimney from site ST07 (15 kV, AsB mode). Note that those were the loci of subsequent mineralization following seawater circulation in the edifice (with 1–3 arrows indicating increasing mineralization steps). The deriving carbonates appeared as needles of aragonite.

**FIGURE 3:**
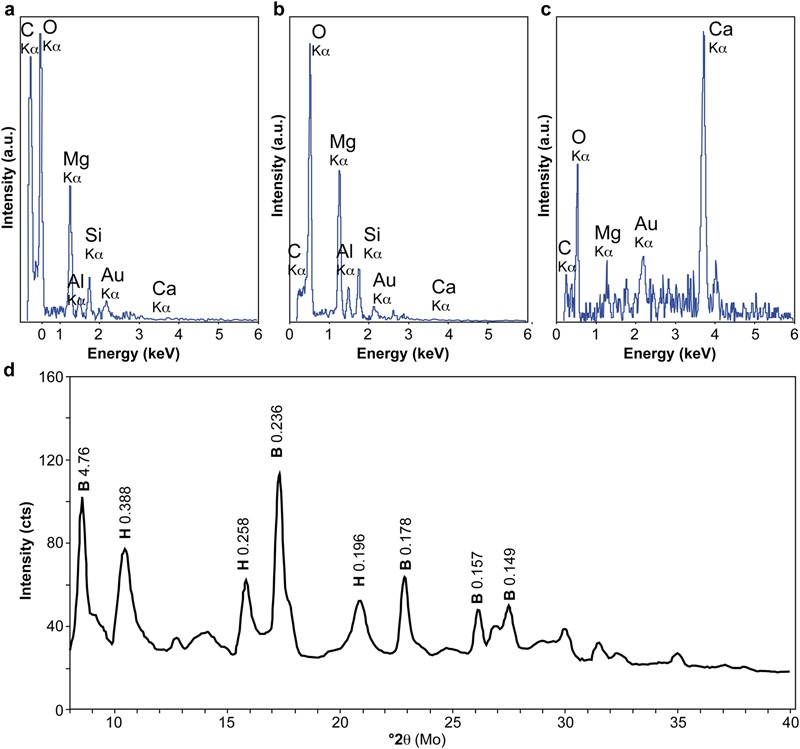
**Scanning Electron Microscopy-Energy Dispersive X-ray spectroscopy (SEM-EDX) and X-Ray Diffraction (XRD) analyses of mineralized filaments from ST09 and ST07 sites. (a–c)** SEM-EDX spectra of a C-rich filament **(a)** and encrusting O- and Mg-rich minerals **(b)** in ST09 samples and of secondary Ca- and O-rich minerals in ST07 samples **(c)**. **(d)** X-ray diffractogram of individualized mineralized biofilament extracted by laser microdissection from an internal conduit sample of ST09 chimney. It showed the predominance of brucite (B) and minor hydrotalcite (H). Main d-spacings (in nm) are notified on the panel.

As shown in **Figures 2a–e**, SEM observations carried out on the young chimney collected at ST09 revealed that the interior of the active conduits was formed by the interweaving of numerous mineralized filaments up to hundreds of micrometer long. Similar encrusted filaments were observed in carbonated hydrothermal chimneys deriving from serpentinization at the LCHF (Kelley et al., 2005) and at the Shinkai Seep Field (Southern Mariana forearc; Okumura et al., 2016). More specifically, Okumura et al. (2016) noted the large presence of mineralized filamentous microorganisms in brucite-dominated chimneys submitted to high fluid discharge conditions that are comparable to the ST09 ones. But, at Shinkai Seep Field, as well as at LCHF, although filaments were assumed to be of bacterial origin, no direct or indirect evidences are provided. At PHF, SEM-EDX analyses showed that these filaments corresponded to C-rich structures (**Figure 3a**) of few micrometers in diameter on which the mineralization process appeared to initiate (**Figure 2a**). These structures were progressively encrusted by round-shaped and flaky or plate-like crystals that increased the filament diameter up to a few tens of μm (**Figures 2b–d**). SEM-EDX spectra collected on numerous minerals encrusting the C-rich filaments were indicative of the presence of Mg and O (**Figure 3b**). This observation was supported by XRD analyses of individual encrusted filaments extracted by laser microdissection that highlighted that the mineral crust was mainly made of brucite (**Figure 3d**). Brucite was sometimes associated with minor hydrotalcite, being either a brucite transformation by-product or a co-precipitated phase (**Figure 3d**). Whereas the hydrothermal fluid was supersaturated with respect to Ca- and Mg-carbonates along with Mg-hydroxides (Monnin et al., 2014), the dominance of brucite, a Mg-hydroxide [Mg(OH)_2_], with minor hydrotalcite, a layered double hydroxide [Mg_6_Al_2_CO_3_(OH)_16_⋅4(H_2_O)], jointly with the absence of carbonates in the most juvenile chimneys bathed by the hydrothermal fluids, suggested that these fluids were depleted in carbonate ions. It also attests to very low CO_2_ partial pressure (pCO_2_) at site ST09, as confirmed by fluid analysis (Supplementary Table S2), even if local variations of the pCO_2_ may have occurred allowing minor hydrotalcite precipitation. Upon brucite precipitation, the filamentous structures became more dense and aggregated, leading to the formation of compact walls formed by cemented, mineralized filaments (**Figure 2e**). Similar tangled filaments were found in the inner conduits of the more mature chimney samples collected at site ST07. These samples were covered by or embedded with secondary carbonates (e.g., aragonite needles), as indicated by SEM-EDX analyses displaying a high Ca and O signal (**Figure 3c**) and XRD analyses, attesting to a greater degree of mixing of hydrothermal fluids with seawater in mature chimneys (**Figure 2f**). Indeed, as stated in Monnin et al. (2014) who emphasized the role of fluid mixing in mineral formation, seawater input is mandatory for carbonate precipitation.

**FIGURE 4:**
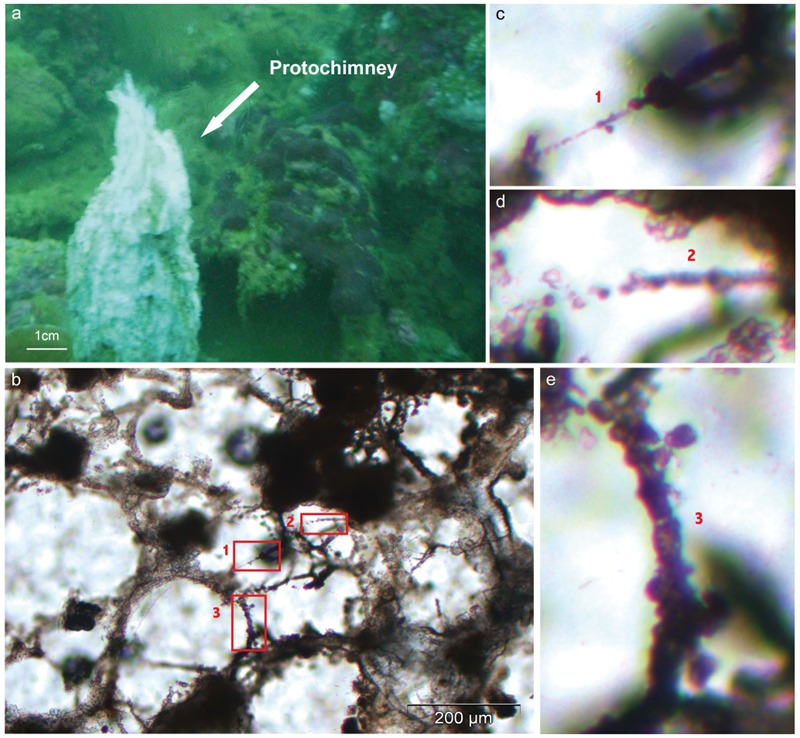
**(a)** Dive photograph of whitish centimetric protochimneys typifying nascent overgrowth on the top of active hydrothermal edifice at the location where the hydrothermal fluids actively discharge (Site ST12; modified from Quéméneur et al., 2014). **(b–e)** Optical views of a thin section obtained from resin embedded protochimneys and showing from the very first instants of the hydrothermal venting the presence of mineralizing filaments [progressive mineralization steps can be followed by the windows numbered from 1 to 3 in **(b)** and magnified in **(c–e)**].

**FIGURE 5:**
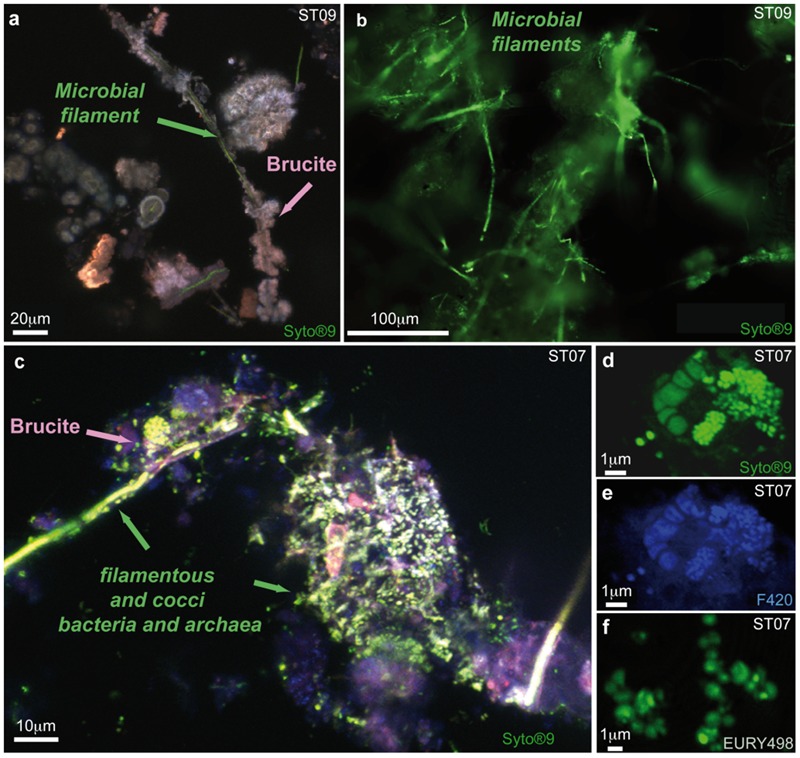
**Epifluorescent image and maximum intensity projection of stacked Confocal Laser Scanning Microscopy (CLSM) images highlighting a different microbial diversity between young ST09 chimney and mature ST07 chimney. (a)** Maximum intensity projection of stacked CLSM images illustrating the quasi exclusive presence of Syto^®^9-stained biofilaments (green) being mineralized by brucite (in pink) in juvenile chimney conduits sampled at site ST09, without any archaeal cell around (no F_420_ autofluorescence); **(b)** Detail of the filament-rich areas observed by epifluorescence after Syto^®^9-staining that were dissected for 16S rRNA gene analysis; **(c)** Composite image showing the presence with respect to brucite mineralization of diverse microorganisms and notably of archaeal cocci (Syto^®^9 fluorescence superimposed with F_420_ autofluorescence) and filaments in ST07 mature chimney; **(d)** Clusters of Syto^®^9-stained cells showing cocci of less than 1 μm in size; they were identified as methane-cycling archaea thanks to the autofluorescence of the F_420_ factor implied in their metabolism **(e)** and to the positive fluorescence after hybridization with the FITC-labeled probe specific for *Euryarchaeota* EURY498 **(f)**.

These C-rich filaments, upon which chimney-forming minerals appeared to nucleate and grow, were ubiquitous, found in all the chimneys sampled in the bay, at the deep sites (Supplementary Figures S3a–d,h–k) as well as at the coastal ones (Supplementary Figures S3e–g). Thus, it seems to represent a general pattern for the construction and the early consolidation of the PHF hydrothermal concretions. Mineralized filaments were also detected from the very early stages of the vent history, notably in the whitish protochimneys that typified nascent overgrowth where the most pristine fluids are discharged (**Figure 4**). This probably indicates that the filaments are likely of subseafloor origin and are brought with the hydrothermal fluids to the seafloor where they begin to mix with seawater, as shown by Monnin et al. (2014) who located the mixing zone not in the subsurface but at the point of discharge. Epifluorescent and CLSM images carried out on ST09, ST11, and ST07 samples stained with Syto^®^9 suggest a biological origin to the C-rich filaments (**Figures 5a–c**). They corresponded to microbial sheaths in which individual rod-shaped cells of few micrometers are visible (**Figure 5b**).

Thermodynamic calculations showed that the fluids at ST09 and ST07 are supersaturated with respect to brucite at pH values higher than 9.5 (Monnin et al., 2014) suggesting that brucite may precipitate abiotically from the fluid. However, the filamentous organisms appear to predominantly impact the concretion formation through organomineralization processes during the earliest stages of the hydrothermal discharge. Organomineralization refers to biologically induced mineralization through the local modification of the chemical environment by microbial activity and/or to passive mineral precipitation in the presence of extracellular polymeric substances (EPS) or biological membranes as nucleation sites (Perry et al., 2007; Defarge et al., 2009). Brucite precipitation has been shown to be induced by the alkalinizing activity of corals, diatoms, algae, or cyanobacteria when coupled to a lowering of CO_2_ partial pressure and an increase of Mg^2+^ activity (Schmalz, 1965; Nothdurft et al., 2005; Buster and Holmes, 2006). However, at PHF, considering the high pH reported in Monnin et al. (2014) for the hydrothermal fluids at ST09 (up to 10.62; Supplementary Table S2), such an alkaline engine does not appear to be the missing motive force in the conduits of the chimney. Conversely, brucite precipitation was experimentally shown to co-occur with organic polymer excretion. Brucite nuclei are stabilized by carboxylate moiety (R-COO^-^) from microbial cell walls at the surface of diatoms, also accelerating the polymerization of larger brucite crystals from Mg(OH)_2_ molecules in water (Sato et al., 2003; Tesson et al., 2008). Accordingly, at PHF, the filamentous microorganisms and their sheaths likely served as nucleation sites for brucite precipitation.

### Subsurface Bacteria as Early Colonizers of Hyperalkaline Venting Systems?

The filamentous organisms thriving in the chimney conduits were identified on several young chimney samples by targeted phylogenetic analyses. PCR amplification, cloning and sequencing of 16S rRNA gene sequences from DNA extracted from filament-rich areas dissected from the inner conduit of ST09 young chimney (**Figures 5a,b**) revealed that the brown microbial mat was mainly dominated by a single bacterial phylotype belonging to the *Firmicutes* phylum (33/41 clones; **Table 1**). The 33 *Firmicutes* clones corresponded to a single OTU (more than 97% identity at the level of their 16S rRNA gene sequences) represented by the clone KM207235 HPst091-1-1. It belonged to a group of uncultivated bacteria that were mainly identified in alkaline and/or subsurface environments (Supplementary Figure S4). The closest environmental sequence (98% identity) of HPst091-1-1 was retrieved from the Donana’s suboxic aquifer (Spain; Lopez-Achilla et al., 2007). The closest cultivated species of HPst091-1-1, *Dethiobacter alkaliphilus* (88% identity), is a facultative autotrophic strain growing with H_2_ as electron donor and S-compounds, but not sulfate, as electron acceptors. It can also use acetate as a carbon source and diverse C-compounds as both electron donors and carbon sources (Sorokin et al., 2008) and is related to sequences commonly obtained from continental serpentinites (Crespo-Medina et al., 2014).

**Table 1:** Phylogenetic affiliations of the representative 16S rRNA gene sequences of the bacterial OTUs (97% similarity) detected following subsampling by dissection of the juvenile chimney conduit collected in ST09 edifice.

OTUs	Closest environmental sequence	16S rRNA gene identities (%)	Isolation source	Taxonomic affiliation	Number of clones/41
HPst091-1-1	DQ837275	98	Pristine coastal aquifer in Donana National Park, Spain	*Firmicutes*	33
HPst09-1-17	AM777997	98	Subterrestrial high pH groundwater associated to serpentinization	*Deinococcus-Thermus*	2
HPst09S-2	KM071636	99	Deep sea hydrothermal vent sediment	*Proteobacteria*	1
HPst09-2-3	GU118133	100	Corals	*Proteobacteria*	5

CLSM observations carried out on the dense biofilm covering the minerals forming the internal conduit of the mature chimney from ST07 revealed a higher diversity of microorganisms highlighted by the green-fluorescence of Syto^®^9-stained filamentous and cocci cells (**Figures 5c,d**). Among the cocci, irregular cells were commonly aggregated into clusters of 1 μm and presented various stages of cell division (**Figures 5c–e**). Notably, they displayed the typical blue-green autofluorescence of the F_420_ cofactor (**Figure 5e**), a key co-enzyme involved in anaerobic methane-cycling (Thauer, 1998). Their affiliation to *Euryarchaeota* was confirmed by FISH (**Figure 5f**). This was in agreement with the phylogenetic analyses of the archaeal diversity carried out at PHF that showed the dominance of the uncultured LCMS in ST07 chimneys (Quéméneur et al., 2014; Postec et al., 2015). Note that these archaeal cocci were never found in the conduits of the young chimney collected at sites ST09 and ST11 (**Figure 5a**). The staining and FISH experiments carried out on ST09, ST07, and ST11 chimneys, combined with 16S rRNA gene sequence analyses on filament-rich areas of the juvenile ST09 chimney, indicated that the dominant OTU KM207235 HPst091-1-1 was part of the early colonizers of the PHF hydrothermal edifices, prior to archaea. The subseafloor origin of these filaments is suggested by their presence in the nascent protochimneys, their role in early chimney construction, and by the affiliation of the OTU KM207235 HPst091-1-1 to sequences retrieved from other subsurface environments.

**FIGURE 6:**
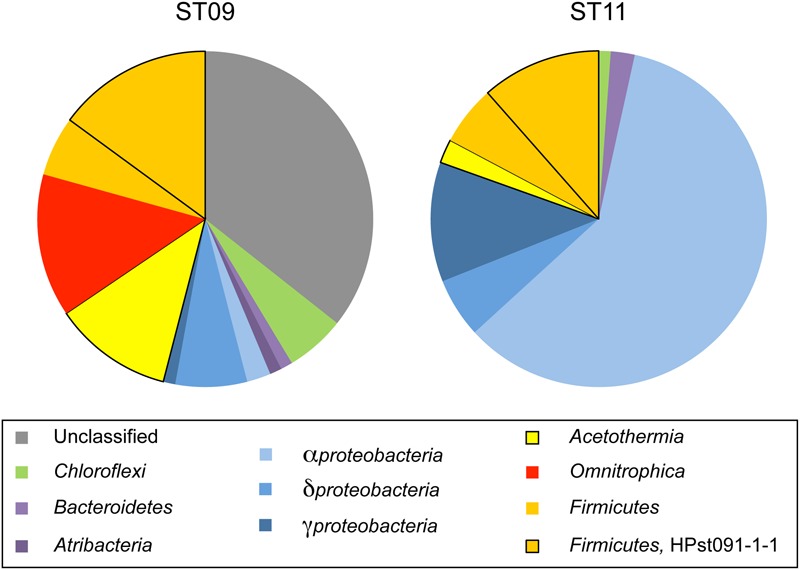
**Pie charts showing the relative proportions of phylum-level affiliated sequences retrieved in juvenile chimneys from sites ST09 and ST11 (bulk chimney samples).** The dominance of *Alphaproteobacteria* in site ST11 attested for highest seawater contamination compared to ST09 chimney whose members are dominated by representatives of deep serpentinisation environments (**Figure 7**). OTU KM207235 HPst091-1-1 represented in both case the major *Firmicutes* species (i.e., 67 and 72% of this phylum sequences for ST11 and ST09, respectively). Taxonomic affiliations were obtained by comparing partial 16S rRNA gene sequences with sequences of the GenBank database using BLAST (Altschul et al., 1997). The closest environmental sequence and cultivated species are detailed in **Figure 7** for site ST09 and in Supplementary Table S3 for site ST11.

**FIGURE 7:**
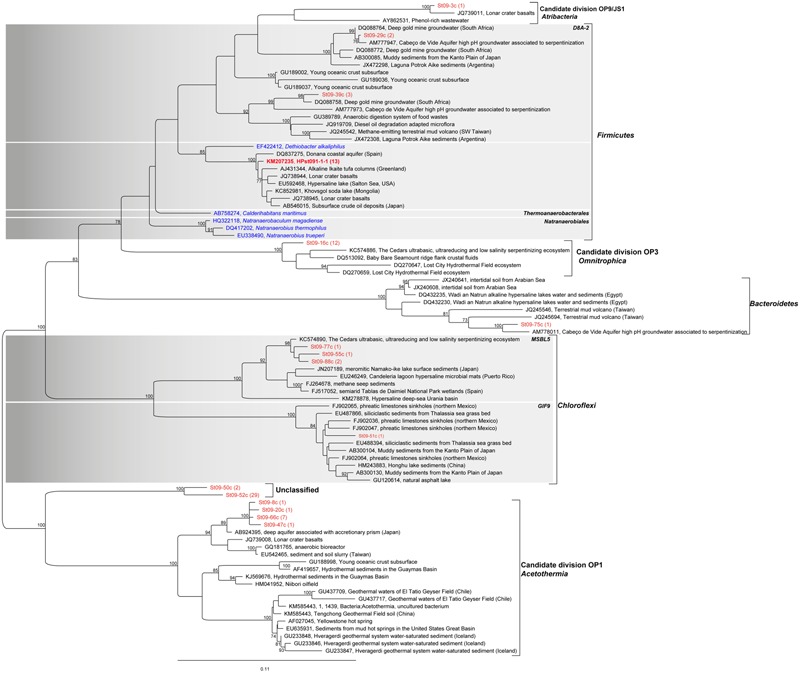
**Phylogenetic tree of 16S rRNA gene sequences of *Firmicutes, Acetothermia, Omnitrophica, Atribacteria, Bacteroidetes*, and *Chloroflexi* (all in red) retrieved in the conduits of the nascent chimney sampled at site ST09 (bulk analysis).** It shows in particular the position of the OTU KM207235 HPst091-1-1 also retrieved from conduit samples observed using CLSM (**Table 1**) and dominating within the *Firmicutes* phylum (**Figure 6**). The closest relatives are for all sequences, uncultured specimens hence, making difficult any assumptions on the involved metabolisms. Note that some sequences were only retrieved at PHB (e.g., St09-84c, St09-52c, St09-50c). The tree was constructed by maximum likelihood analysis, using 1,210 positions, including the closest possible uncultivated (black) and cultivated (blue) relatives as well as more distant representatives of cultivated species. Bootstrap values for nodes (>70% support) based on 1,000 replicates are displayed as percentages. The number of retrieved sequences is indicated in brackets after each clone name.

In order to assess the representativeness of the bacterial filaments retrieved in the dissected conduit samples, bacterial diversity was characterized from DNA extracted from bulk rock samples bathed by the hydrothermal fluid in both ST09 and ST11 chimneys (**Figure 6**; Supplementary Table S3). Congruent with the lack of detection of methanogenic archaea by microscopic examination, we did not detect any archaeal 16S rRNA gene sequences by PCR amplification. As shown in **Figure 6**, the community of the inner conduits depicted by cloning and Sanger sequencing confirmed that *Firmicutes* were important inhabitants of ST09 and ST11 bulk chimney conduits (representing 20.7 and 17.2% of all retrieved sequences, respectively). It is worth noting the apparent discrepancy between the two sequencing approaches (i.e., targeted filament-rich areas *vs.* blindly bulk sampling), notably the fact that KM207235 HPst091-1-1 was not the dominant OTU when DNA was extracted from bulk chimney samples. This may be due to its thick, mineralized sheath that may hamper cell lysis. Nevertheless, the *Firmicute*s phylum was interestingly dominated by the KM207235 HPST091-1-1 sequence, representing 14.8 and 11.5% of all sequences retrieved for ST09 and ST11, respectively, (i.e., 72 and 67% of the total *Firmicutes*). Note in addition that this *Firmicutes* species was not restricted to these two juvenile chimneys and similar sequences were also reported under the accession numbers KF886161 (clone PHFST07_B16) and KF886087 (clone PHFST08_B19) in ST07 and ST08 sites (Supplementary Figure S1; Quéméneur et al., 2014) and under the accession number KJ159205 (clone PHF_15-B34_D22) in other chimneys of site ST09 (Postec et al., 2015). As shown in **Figure 7** and Supplementary Table S3 and with the sole exception of ST11d-17, the other sequences affiliated to the *Firmicutes* phylum in ST09 and ST11 related to sequences retrieved from other chimneys from PHF ST09 site (Postec et al., 2015), in the serpentinization-driven subterrestrial alkaline aquifer of Cabeço de Vide aquifer in Portugal (Tiago and Verissimo, 2013), in a deep H_2_-rich gold mine in the South Africa Precambrian craton (Lin et al., 2006) and in a terrestrial hydrocarbon seep in Taiwan (Cheng et al., 2014).

In the bulk chimney samples collected in ST09 conduits, we detected numerous sequences of *Acetothermia* (candidate division OP1) and *Omnitrophica* (candidate division OP3) (Hugenholtz et al., 1998; Rinke et al., 2013), respectively, representing 11.5 and 13.8% of all retrieved sequences. Similar to *Firmicutes*, these bacteria may also constitute key components of the ST09 community. The sequences affiliated to *Acetothermia* were related to taxa retrieved from a deep aquifer showing high methane production (Kimura et al., 2010). The closest relatives of the sequences affiliated to *Omnitrophica* were retrieved from a subseafloor environment along the Juan de Fuca Ridge flank (Huber et al., 2006), which are also closely related to sequences from the Cedars (Suzuki et al., 2013) and Lost City (Brazelton et al., 2006) hydrothermal fields. Additionally, as shown in **Figure 7**, a large number of unclassified sequences (up to 35.6% of the total sequences; **Figure 6**) were also related to *Acetothermia* (St09-52c, St09-50c). Nonetheless, they only shared 87% identity with their closest environmental sequence retrieved in a hydrothermal system associated with a back-arc seated seamount (Stott et al., 2008). Interestingly, Postec et al. (2015) has reported the occurrence of similar but shorter sequences forming a distinct clade during the 6-year monitoring of ST09 site. Both *Acetothermia* and *Omnitrophica* affiliated sequences occurred at a level comparable to the present study (each representing 13.8% of the total clones). The sequences retrieved in our study from bulk samples collected in ST11 juvenile chimneys (**Figure 6**; Supplementary Table S3) indicated that *Alphaproteobacteria* dominated the bacterial community (i.e., 59.8% of all retrieved sequences; **Figure 6**), thus providing evidence for more important mixing with seawater compared to ST09 chimney. Members of *Acetothermia* were still detectable but to a much lesser extent (i.e., 2.3% of all sequences) and no members of *Omnitrophica* were highlighted. Although *Proteobacteria* were less abundant in ST09 chimney conduits (10.3% of all sequences compared to 77.0% for ST11), the *Deltaproteobacteria* constituted the most important class (6.9% of all sequences or 66.7% of the *Proteobacteria* sequences). Phylogenetic affiliations of the *Proteobacteria* showed that they were related to sequences retrieved in alkaline environments including serpentinization associated ones (Supplementary Figure S5).

These results obtained on bulk chimney samples confirmed the presence of OTU KM207235 HPst091-1-1 retrieved from the filament-rich areas collected in ST09 chimney samples. The predominance of representatives of the *Firmicutes* phylum dominated at ∼70% by one species (KM207235 HPst091-1-1), combined with the absence of seawater species (**Figures 6** and **7**), reinforced the idea that it likely represents one of the microorganisms that first colonized these chimneys and could be representative of community associated with the most pristine hydrothermal fluids. Considering that the bacterial mineralizing filaments were involved in the early stages of chimney construction and that their phylogenetic affiliations suggest a subsurface origin, we conclude that this *Firmicutes* species was discharged with hydrothermal fluids from the subseafloor. The hydrothermal origin of these bacteria may also be assumed based on the fact that the closest environmental sequences of some of the sequences retrieved, including those affiliated to *Gammaproteobacteria, Deltaproteobacteria* (Supplementary Figure S5), *Omnitrophica*, and *Acetothermia* (**Figure 7**) were found in alkaline and/or hot crustal environments. All these taxa may be representative of endolithic ecosystems relying on the serpentinization reaction at depth and thus of SLIMEs. Furthermore, the predominance, during the early stages of chimney construction, of *Bacteria* rather than archaeal *Methanosarcinales* as observed in Lost City (Schrenk et al., 2004; Brazelton et al., 2006), raises questions about the functioning of this serpentinization-related ecosystem.

### Perspectives about the Functioning of Serpentinization-Based Subsurface Ecosystems

Our micrometric scale imaging of the chimney conduits coupled to 16S rRNA gene analyses highlight the importance of bacteria with a subseafloor hydrothermal origin in the serpentinization-related ecosystem of the PHF. In accordance, their metabolisms may document the functioning of a SLIME. However, inferring metabolisms or carbon substrates is particularly challenging if not impossible in the present case as the retrieved taxa are lacking cultivable relatives and are far from any environmental clones (**Figure 7**; **Table 1**; Supplementary Table S3). Nonetheless, due to the potential that they represent a relevant model for the study of serpentinization-based ecosystems, they deserve further attention and efforts.

Members of *Acetothermia* and *Omnitrophica* have been shown to occur across a geochemically diverse range of environments including hydrothermal fields from oceanic ridges (e.g., Brazelton et al., 2006; Perner et al., 2007) to organic-rich environments (Dojka et al., 1998; Teske et al., 2002; Dhillon et al., 2003; Alain et al., 2006; Inagaki et al., 2006; Duncan et al., 2009; Harrison et al., 2009; Kobayashi et al., 2012; Zhou et al., 2012) and also in fluids associated with actively serpentinizing rocks in the Oman ophiolite (Miller et al., 2016). Within the past few years, two nearly complete genomes of *Acetothermia* were established based on cultivation-independent genome-resolved metagenomic analysis of a subsurface thermophilic microbial mat community (Takami et al., 2012) and of an oil reservoir community (Hu et al., 2016). Whereas the first pointed to its capability of implementing acetogenesis through the ancient reductive acetyl-CoA pathway by utilizing CO_2_ and H_2_, the second suggested significant differences in metabolic capacities and was more in favor of heterotrophy with consequences on the biogeochemical transformations occurring in the oil reservoir. In either case, both are very distantly related to the sequences retrieved in ST09 and ST11 chimneys, and the metabolic capacities of taxa from *Acetothermia* and *Omnitrophica* remain entirely unknown at PHF, although their relative proportions and ubiquity may imply a prominent role in the functioning of the ecosystem.

A large diversity of *Firmicutes* was observed at Lost City (Brazelton et al., 2006). When compared to subterrestrial sites of active serpentinization, also rich in electron donors such as H_2_ and CH_4_, bacterial phylotypes belonging to the *Clostridia* class were also found to be dominant and closely related to *D. alkaliphilus* (**Figure 7**; Supplementary Figure S4). This was the case for the deep terrestrial groundwaters at The Cedars formation (Suzuki et al., 2013), Cabeço de Vide aquifer (Tiago and Verissimo, 2013), and Tablelands ophiolitic complex (Brazelton et al., 2012). Molecular microbial surveys of the PHF chimneys have also demonstrated that *Firmicutes* were particularly abundant among the diverse bacterial community (Quéméneur et al., 2014; Postec et al., 2015). The systematic presence of these phylotypes may thus be a common feature of subsurface ecosystems in serpentinization-based environments, as proposed by Suzuki et al. (2013).

It has been highlighted at LCHF and in the mature chimneys of PHF that *Methanosarcinales* were abundant and associated with sulfate-reducing bacteria (SRB). At LCHF, *dsrB* genes, a molecular marker for SRB, affiliated to *Firmicutes* were found to be abundant (Brazelton et al., 2006; Gerasimchuk et al., 2010). In contrast, at PHF, the *dsrB* gene abundance estimated using quantitative PCR indicated that SRB represented less than 6% of the total bacterial community in various ST09 chimneys (Postec et al., 2015) and only 2–8% in other chimneys of the PHF (Quéméneur et al., 2014). Furthermore, *dsrB* genes sequences were affiliated to *Desulfovibrionales* and *Desulfobacterales* orders and not to *Firmicutes* (Quéméneur et al., 2014; Postec et al., 2015). The observation that *Firmicutes* may be one of the first inhabitants of the conduits of the PHF nascent vents, dominated by hydrothermal fluids circulation over seawater, raises the questions of their metabolic capabilities, the nature of the available electron donors/acceptors and carbon sources, as well as their role in the PHF ecosystem, particularly with respect to *Methanosarcinales* that are only observed in more mature chimney conduits. Archaea cycling methane are acknowledged to be highly pervasive across diverse ecosystems, showing a tolerance to wide ranges of extreme physical and chemical parameters, and large ranges of pH (from 3 to 10), temperatures (from 0 to 122°C), and salinities (i.e., 1–5 M NaCl; Hoehler et al., 2010). On the contrary, compared to members of *Firmicutes* that can tolerate oxygen at a relatively high level (Whitham et al., 2015), *Methanosarcinales* are highly sensitive to oxygen (Zinder, 1993; Hoehler et al., 2010). Even if the fluids circulating within juvenile conduits were highly dominated by anoxic hydrothermal fluids, we cannot exclude slight oxygen contamination of the deep fluids when they reached the seafloor, hence affecting *Archaea*. However, DIC concentrations are quite low in the most juvenile ST09 chimney (Supplementary Table S2). It may limit the presence of autotrophic microorganisms. Deprivation in inorganic carbon in the pristine fluids is also indicated by the near absence of solid carbonates in the chimney conduits bathed with the reduced hydrothermal fluids where brucite, a Mg-hydroxide, and minor hydrotalcite, a double-layered Mg-Al-hydroxide, precipitated on the *Firmicutes* filaments (**Figure 3**). As we have no information on the metabolism of the bacteria represented by OTU HPSt091-1-1, we cannot exclude that this strain could be an autotrophic *Firmicutes* consuming the scarce inorganic carbon and preventing the formation of carbonate minerals. Knowing that in the subsurface serpentinizing environments, organic compounds are likely not as limited as the inorganic compounds (Schrenk et al., 2013), we propose an alternative hypothesis that could explain the predominance of *Firmicutes* over *Methanosarcinales* in juvenile chimney conduits. Organic carbon compounds can indeed have multiple origins including *in situ* microbial activities at depth (Ménez et al., 2012), hydrothermal degradation of biological remnants (Pasini et al., 2013) and abiogenic synthesis whose amplitude and pathways still need to be assessed in natural systems (Sephton and Hazen, 2013). Consistently, heterotrophic and fermentative species belonging to the *Firmicutes* phylum have been retrieved from active serpentinization sites (Brazelton et al., 2012; Schrenk et al., 2013; Tiago and Verissimo, 2013). Notably, the two obligate alkaliphilic strains recently isolated from the interiors of PHF chimneys (*Alkaliphilus hydrothermalis* and *Acetoanaerobium pronyense*; Ben Aissa et al., 2014; Bes et al., 2015) can produce H_2_, CO_2_, and acetate from the fermentation of various carbon sources or the disproportionation of crotonate to acetate and butyrate (Ben Aissa et al., 2014). Hence, as proposed by Schrenk et al. (2013) heterotrophy and fermentation may be important metabolic strategies in these serpentinizing environments where autotrophy is severely challenged by the limited availability of inorganic C-compounds, those being instead very rapidly trapped into solid carbonates at these hyperalkaline pH (Alt and Teagle, 1999; Bradley et al., 2009). Lever (2012) has shown that under conditions similar to those encountered at the PHF, the versatility of the biochemical pathways of heterotrophic microorganisms such as acetogens may confer to them an ecological advantage compared to the more substrate specialized *Methanosarcinales*. This might be supported by a preliminary study carried out on the PHF fluids and showing elevated acetate concentrations (around 70 μM) and minor formate (3–4 μM) whereas inverse proportions were found at LCHF (8 and 140 μM for acetate and formate, respectively; Lang et al., 2010). We thus hypothesize that the dominant bacteria associated with the serpentinizing fluids in the early stages of chimney formation at PHF could have versatile metabolisms that would allow them overcoming the scarcity of inorganic carbon compounds.

Considering the potential of the present taxa to be representative members of serpentinization-based ecosystems and in order to infer their metabolic capabilities, genomic analysis from single cell isolates along with metagenomic analyses could prove highly enlightening. Similarly, a better understanding and consideration of the diversity of carbon sources in the serpentinizing subsurface could shed more light on the way they shape microbial ecosystems at depth. Finally, considering that serpentinization-based ecosystems are considered as analogs of primitive microbial ecosystems, and that some authors suggest that *Acetothermia* may be deeply branched on the tree of life (Takami et al., 2012), elucidating the metabolic capabilities of the dominant bacteria at PHF may also give clues about ancestral metabolisms.

## Author Contributions

EG, MG, BM, and CP made the analyses, discussed the results, and wrote the paper. SQL performed the organic compounds analyses. All others authors participated to the PHF sampling, commented the results, and co-wrote the paper.

## Conflict of Interest Statement

The authors declare that the research was conducted in the absence of any commercial or financial relationships that could be construed as a potential conflict of interest.
