# Supplementary material for: Mineralizing Filamentous Bacteria from the Prony Bay Hydrothermal Field Give New Insights into the Functioning of Serpentinization-Based Subseafloor Ecosystems
Source: Front Microbiol. 2017 Jan 31;8:57. doi: 10.3389/fmicb.2017.00057 (PMC5281578; doi:10.3389/fmicb.2017.00057)
Supplement: Supplementary file 1 [file Image_1.pdf]

Supplementary Material

Mineralizing filamentous bacteria from the Prony Bay Hydrothermal Field give new insights into the functioning of serpentinization-based subseafloor ecosystems

Céline Pisapia [1,2]*, Emmanuelle Gérard [1], Martine Gérard [3], Léna Lecourt [1], Susan Q. Lang [4], Bernard Pelletier [5], Claude E. Payri [6], Christophe Monnin [7], Linda Guentas [6,8,9,10], Anne Postec [11], Marianne Quéméneur [11], Gaël Erauso [11], Bénédicte Ménez [1]*

[1] Institut de Physique du Globe de Paris, Sorbonne Paris Cité, Université Paris Diderot, CNRS, Paris, France

[2] Synchrotron SOLEIL, DISCO beamline, Saint Aubin, France

[3] Institut de Minéralogie, de Physique des Matériaux et de Cosmochimie, Institut de Recherche pour le Développement, Université Pierre et Marie Curie, Paris, France

[4] School of the Earth, Ocean and Environment, University of South Carolina, Columbia, USA

[5] GIS Grand Observatoire de l'environnement et de la biodiversité terrestre et marine dans le Pacifique Sud, Centre IRD de Nouméa, Nouméa, New Caledonia

[6] UR227 COREUS, Centre IRD de Nouméa, Nouméa, New Caledonia

[7] Géosciences Environnement Toulouse, Univ Paul Sabatier/CNRS/IRD, Toulouse, France

[8] Université de Toulon, Laboratoire Matériaux Polymères Interfaces Environnement Marin EA 4323, La Garde, France

[9] MIO, Centre IRD de Nouméa, Nouméa

[10] Université de la Nouvelle-Calédonie, LIVE, BPR4, Nouméa, New Caledonia

[11] Aix Marseille Université, CNRS/INSU, Université de Toulon, IRD, Mediterranean Institute of Oceanography (MIO), UM 110, 13288 Marseille, France

*** Correspondence:**

Pisapia Céline, Geomicrobiology group, IPGP, Sorbonne Paris Cité, Université Paris Diderot, CNRS, Paris, France, celine.pisapia@gmail.com

Ménez Bénédicte, Geomicrobiology group, IPGP, Sorbonne Paris Cité, Université Paris Diderot, CNRS, Paris, France, menez@ipgp.fr

1. Supplementary Figures

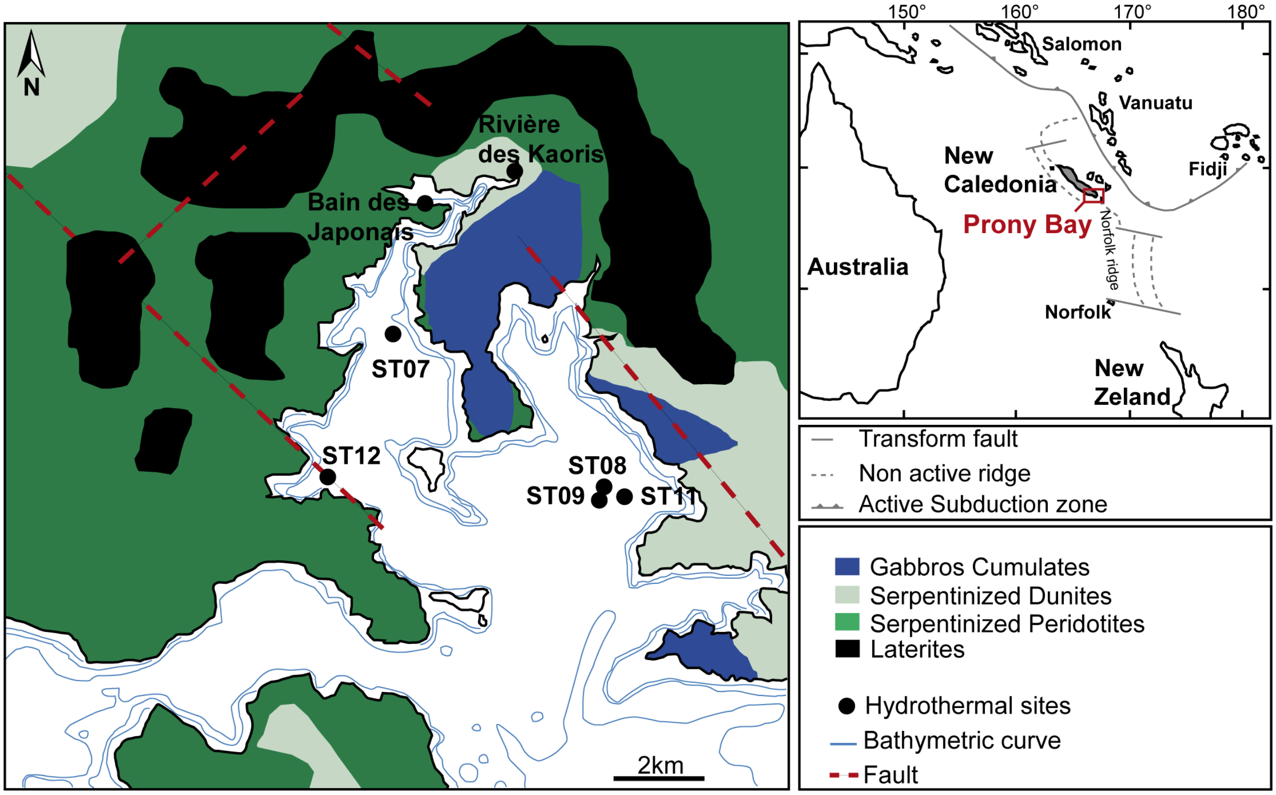

Figure S1. Simplified geological map and bathymetric curves of the Prony Bay, New Caledonia, South Pacific (modified from Monnin et al., 2014)**.** The Western coast of the bay is constituted by serpentinized peridotites while the Eastern coast corresponds to gabbros and dunites. The geological substrata of the Prony Bay hydrothermal field are then comparable to those of the Lost City hydrothermal field (Kelley et al., 2001). Coastal and lagoonal hydrothermal vents, including sites ST09, ST11 and ST07 specifically studied here, are localized with black dots. They were visited and sampled since 2005, especially in 2011 during the French HYDROPRONY cruise on the *R/V Alis* (Pelletier et al., 2011). Further description can be found in Monnin et al. (2014), Quéméneur et al. (2014) and Postec et al. (2015).

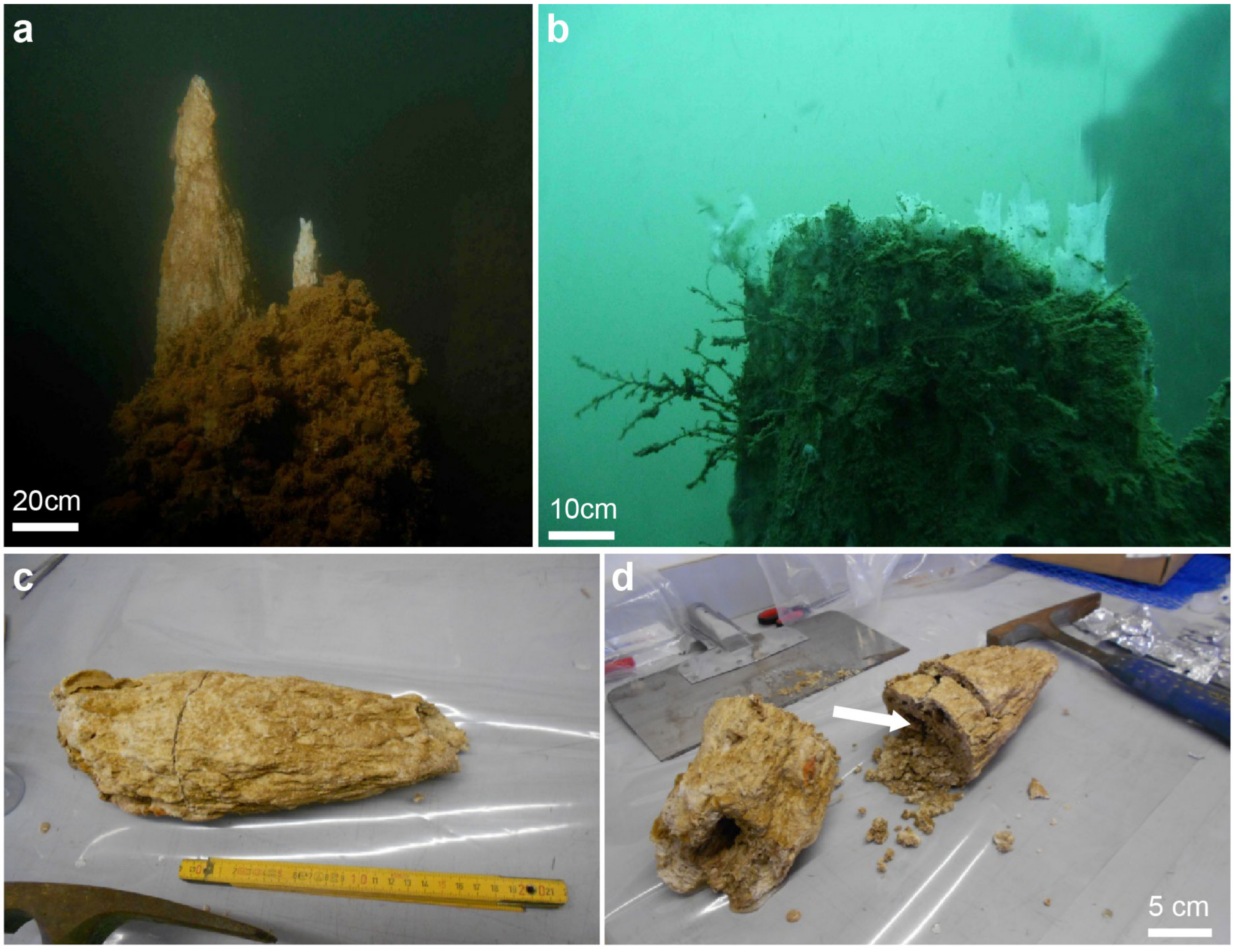

Figure S2. Photographs of the ST11 hydrothermal edifices from the Prony Bay (New Caledonia) characterized in the present study and sampled by scuba divers in 2011: **(a)** and **(b)** are dive photographs of ST11 carbonated chimneys hosted at 47 mbsl showing a consolidated and likely ancient base topped with juvenile chimneys and small white juvenile protochimneys where hydrothermal fluids are discharged. **(c)** and **(d)** are detailed photographs of the young chimney sampled with transversal cut (d) showing the internal hydrothermal conduit (white arrow).

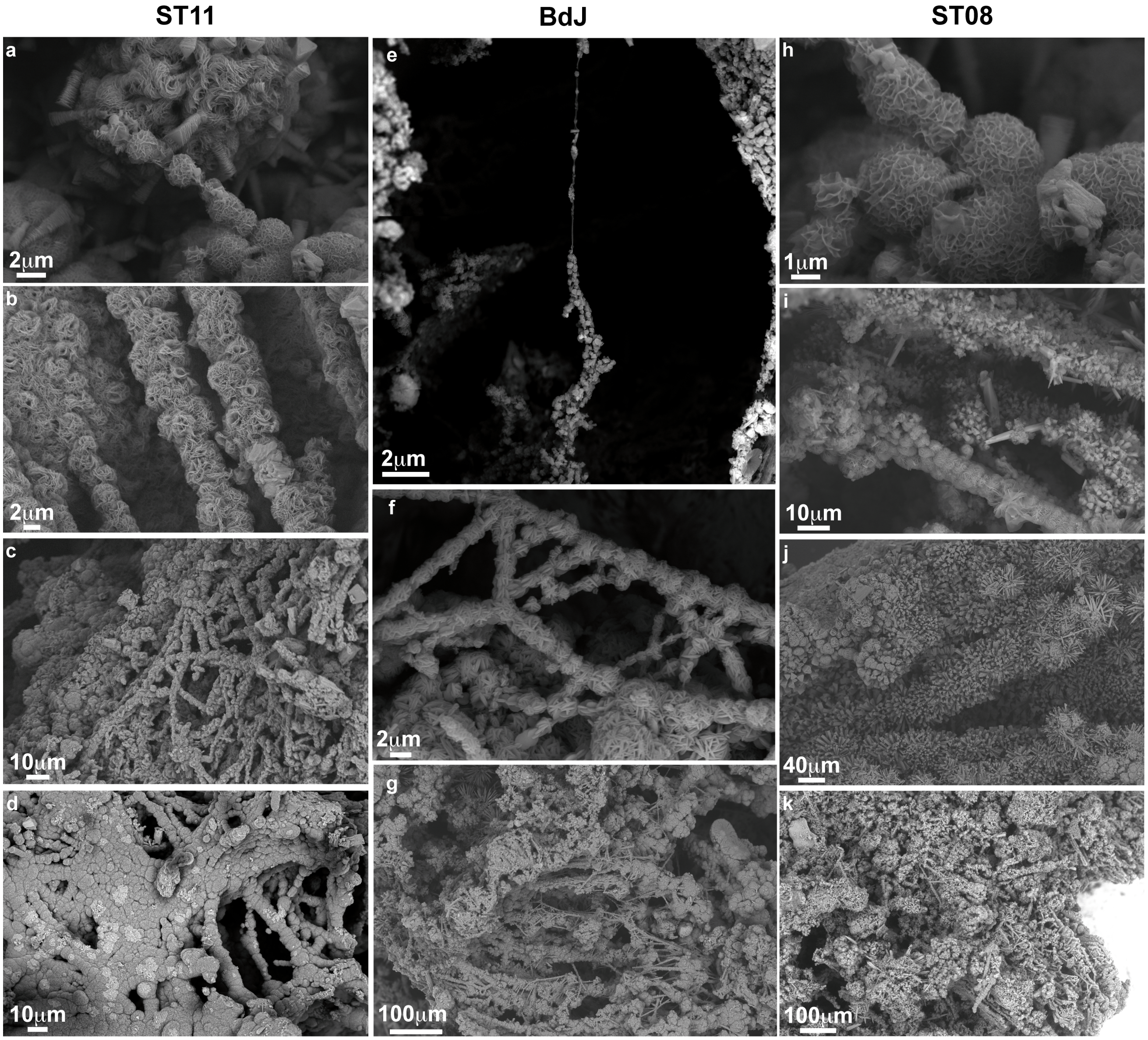

Figure S3. SEM images in AsB mode collected on hydrothermal chimneys sampled in various sites of the Prony Bay Hydrothermal Field: ST08 [**(a)** to **(d)**, at 8kV for **(b)** and **(c)** and 10 kV for **(a)** and **(d)**], ST11 [**(e)** to **(g)**, at 15 kV], and BdJ for Bain des Japonais [**(h)** to **(k)**, at 10 kV for **(h)** and **(j)** and 15 kV for **(i)** and **(k)**]; see Supplementary Figure S1 and Supplementary Table S1. They indicate that the filament mineralization is common to all sampled vents, hence leading to consider this process as a general mechanism in early chimney construction at this hyperalkaline hydrothermal field.

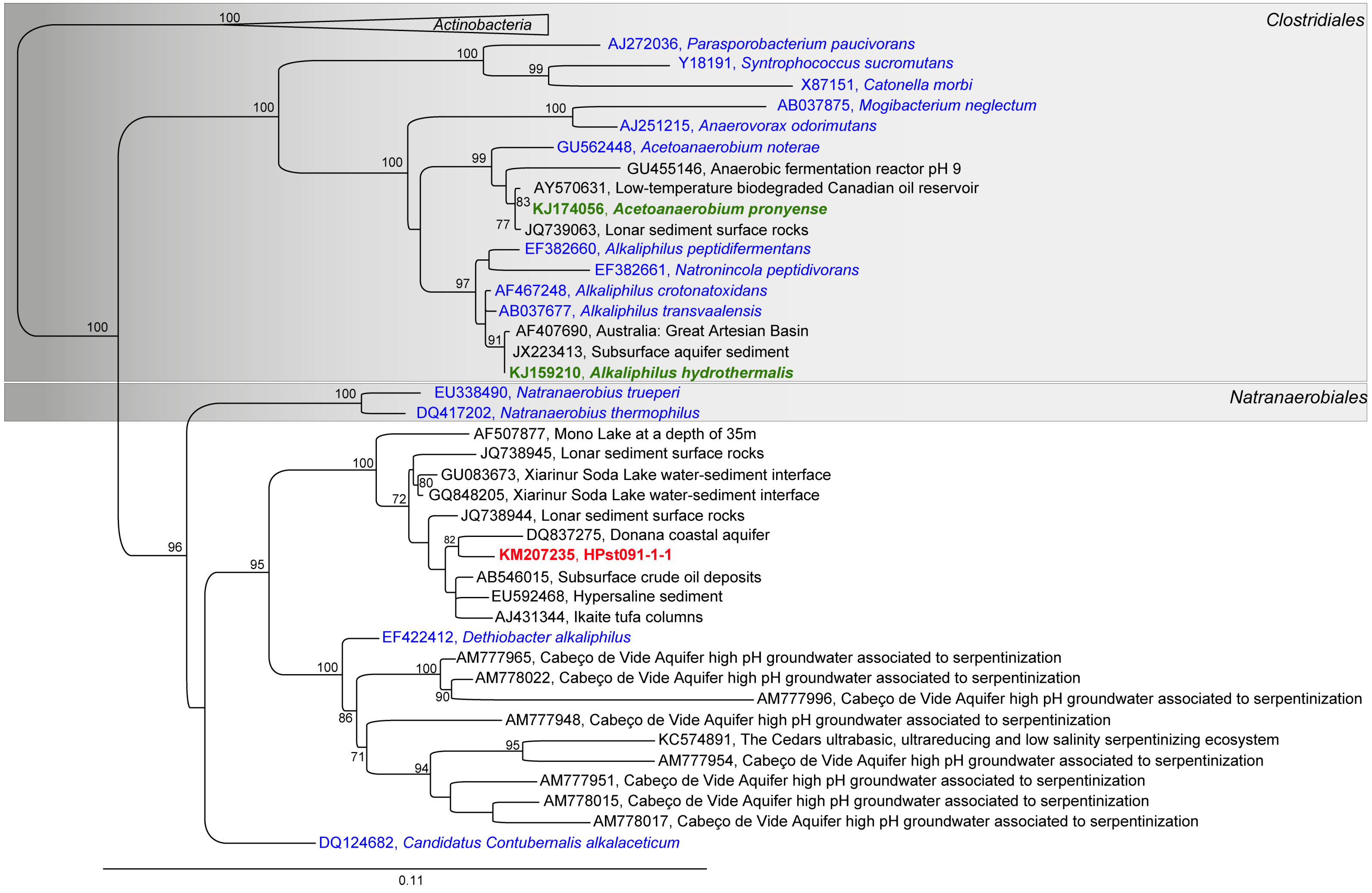

Figure S4. Phylogenetic tree of 16S rRNA gene sequences showing the position of the filamentous *Firmicutes* (OTU HPst091-1-1 in red) systematically identified in the conduits of the PHF nascent chimneys (Table 1). The tree was constructed by maximum likelihood analysis, using 895 positions, including the closest possible uncultivated (black) and cultivated (blue) relatives as well as more distant representatives of cultivated *Firmicutes*. The OTU HPst091-1-1 sequence was notably aligned with two sequences of *Firmicutes* strains (*A. hydrothermalis* and *A. pronyense,* accession numbers KJ159210 and KJ626326, respectively; Ben Aissa et al., 2014; Bes et al., 2015) recently isolated from PHF chimneys (in green). Bootstrap values for nodes (>70% support) based on 1,000 replicates are displayed as percentages.

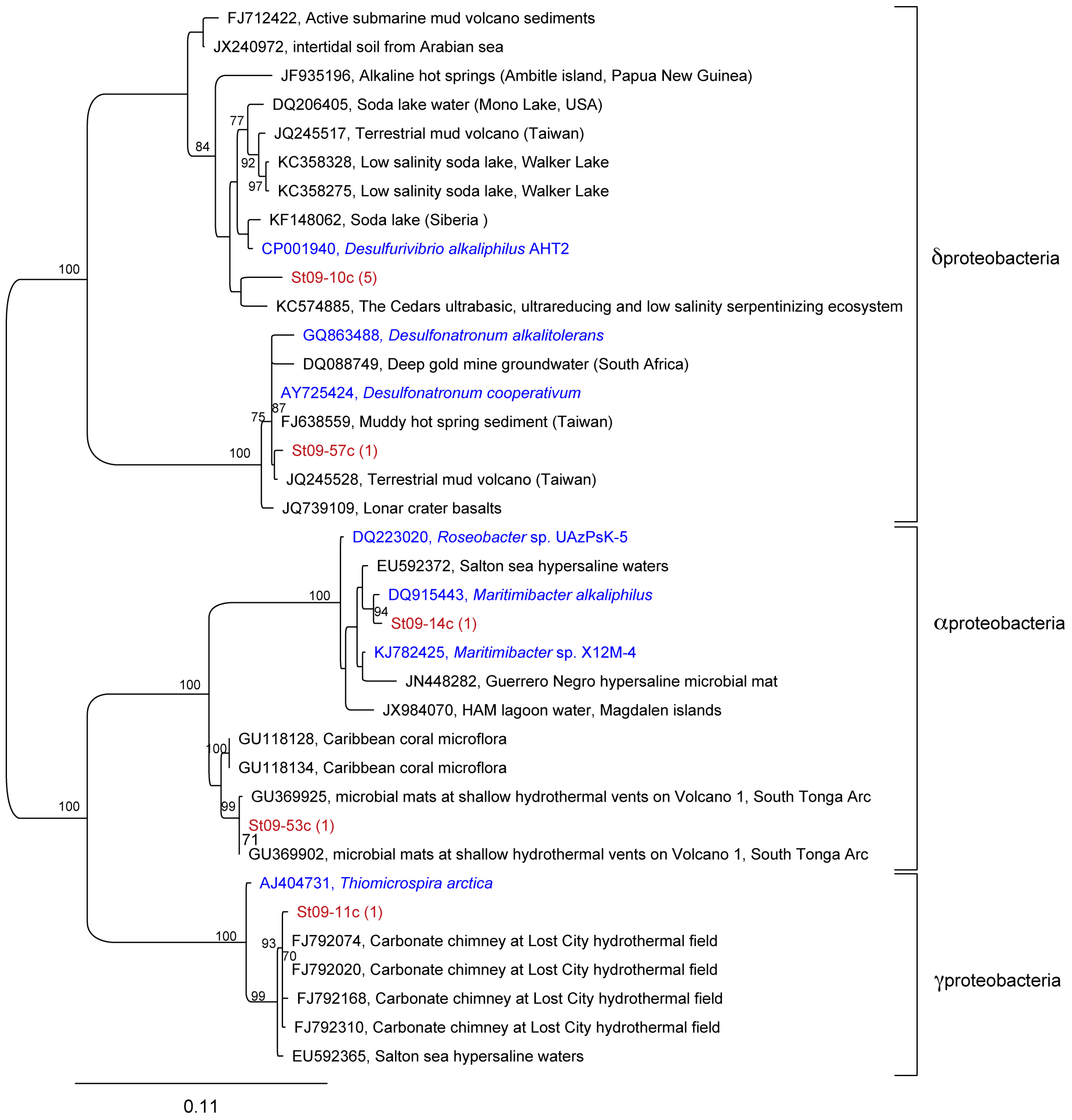

Figure S5. Phylogenetic tree of 16S rRNA gene sequences of *Proteobacteria* (in red) retrieved in the conduits of the nascent chimney sampled at site ST09 (bulk). The tree was constructed by maximum likelihood analysis, using 945 positions, including the closest possible uncultivated (black) and cultivated (blue) relatives as well as more distant representatives of cultivated species. Bootstrap values for nodes (>70% support) based on 1,000 replicates are displayed as percentages. The number of retrieved sequences is indicated in brackets after each clone name.

2. Supplementary Tables

Table S1. Synthetic table of the studied sites and of the mineralogical, morphological, microscopic and phylogenetic analyses performed.

Site studied	Site depth (mbsl*)	Sampled hydrothermal chimney maturity	Mineralogical and morphological analyses	Epifluorescence and CLSM analyses	FISH experiments**	16S rRNA gene cloning and sequencing
ST07	25	mature and active	SEM - XRD	Syto®9 - F_{420}	EURY498 - CREN499	-
ST08	47	mature and active	SEM - XRD	Syto®9 - F_{420}	-	-
ST09	50	young and active	SEM - XRD	Syto®9 - F_{420}	-	Filament-rich areas - Bulk
ST11	47	young and active	SEM - XRD	Syto®9 - F_{420}	-	Bulk
ST12	38	nascent protochimney	Optical microscopy	-	-	-
Bain des Japonais	< 1	young and active	SEM - XRD	-	-	-

* mbsl : meters below sea level
** Probes were specific for *Euryarchaeota* (EURY498) and *Crenarchaeota* (CREN499). Details can be found in Table S3.

Table S2. Selected elemental compositions and temperatures of the fluids collected at vents from ST07, ST09 and ST11 sites at the Prony Bay Hydrothermal Field, compared with the Lost City Hydrothermal Field and The Cedars (bdl: below detection limit; DIC: Dissolved Inorganic Carbon).

Site	Sample	pH	T(°C)	DIC ($mmol·L^{-1}$)	Ca ($mmol·L^{-1}$)	Mg ($mmol·L^{-1}$)	SO_4 ($mmol·L^{-1}$)	H_2 ($mmol·L^{-1}$)	CH_4 ($mmol·L^{-1}$)	References
Prony Bay Hydrothermal Fluids										Monnin et al. (2014)
**ST07**	W1	9.73		0.5830	4.91	23.32	12.24			
	W2	9.66		0.6666	4.96	22.92	12.14			
	W3	9.67		0.6721	4.43	18.69	10.17			
	W3Ti	9.44		0.6870	7.16	41.14	19.95			
	W4	9.61		0.6342	4.59	20.42	10.82			
	W5	9.72		0.7691	5.17	24.42	12.78			
	W6	9.61		0.7067	5.74	28.73	14.59			
	W7	10.00		0.5377	5.46	27.95	14.20			
	W8	10.13		0.4945	4.41	20.08	10.64			
	W9	9.91		0.4971	5.76	28.36	15.19			
	W10	10.14		0.4482	3.49	12.43	7.53			
	W11	9.96		0.5121	5.45	28.02	14.25			
**ST09**	W1	10.45		0.5280	2.95	3.6	3.31			
	W3	10.62		0.3985	2.76	2.768	1.72			
	W4	10.46		0.4094	4.80	4.8	3.82			
	W6	9.18		1.6885	38.77	38.77	18.85			
	W7	10.51		0.6342	4.96	4.96	4.03			
**ST11**	W1	10.64		0.5376	2.28	2.52	1.54			
	W4	9.58		1.1156	7.28	31.69	16.16			
	W5	8.76		1.8450	9.45	45.44	22.56			
	W10	9.06		1.5244	7.19	31.37	15.79			
	W11	9.38		1.2131	6.31	22.75	12.13			
Lost City Hydrothermal Fluids		9-9.8	40-75	--	21.0-23.3	9-19	5.9-12.9	0.25-0.43	0.13-0.28	Kelley et al. (2001)
The Cedars		11.5-11.9	16-18	0.006-0.035	bdl-0.064	0.93-1.43	bdl-0.001	15.7-50.9 (%/vol)	5.3-15.8 (%/vol)	Suzuki et al. (2013)

Table S3. Phylogenetic affiliations of the 16S rRNA gene sequences of representative bacterial OTUs (99% similarity) detected in the seawater-contaminated chimney conduit collected in the ST11 edifice. Taxonomic affiliations were obtained by comparing partial environmental 16S rRNA gene sequences with sequences of the GenBank database using BLAST (Altschul et al., 1997). ND: not determined.

Clone	Number of retrieved sequences	Closest uncultivated bacteria	16S rRNA gene identities (%)	Closest cultivated bacteria or single cell isolate (SCI)	16S rRNA gene identities (%)	Taxonomic affiliation
St11d-85	1	AB305511 hydrothermal sediment	99	ND		*Alphaproteobacteria*
St11d-74	1	AY989257 uncultured soil bacterium	90	ND		*Alphaproteobacteria*
St11d-32	9	FJ594819 $CaCO_3$ deposition on a metallic artificial reef, Eilat	99	NR_109524 *Marivita geojedonensis*	99	*Alphaproteobacteria*
St11d-82	1	FJ952817 coral-associated bacteria	98	KR347220 *Octadecabacter* sp. CQB-13	98	*Alphaproteobacteria*
St11d-39	2	GU369902 shallow hydrothermal vent region on Volcano 1, South Tonga Arc	99	ND		*Alphaproteobacteria*
St11d-86	2	JF431973 marine biofilm	99	AM180476 *Roseobacter* sp. Ber2107	97	*Alphaproteobacteria*
St11d-91	1	JF952817 coral-associated bacteria	99	KR347220 *Octadecabacter* sp. CQB-13	99	*Alphaproteobacteria*
St11d-30	7	KF886078 Prony Bay hydrothermal carbonate chimney	99	AJ132383 *Natronohydrobacter thiooxidans*	98	*Alphaproteobacteria*
St11d-1	1	KF886079 Prony Bay hydrothermal carbonate chimney	100	AP014648 *Methyloceanibacter caenitepidi*	99	*Alphaproteobacteria*
St11d-41	19	KF886120 Prony Bay hydrothermal carbonate chimney	99	FN811291 *Rhodobacter* sp. p1rd1	99	*Alphaproteobacteria*
ST11-5	1	KJ004401 Hot Lake microbial mat	99	NR_042212 *Roseibaca ekhonensis* strain EL-50	98	*Alphaproteobacteria*
St11d-24	3	KR911716 St09-2-3 this study	99	KJ732892 *Labrenzia sp. CUA-809*	99	*Alphaproteobacteria*
St11d-8	3	KT720453 marine microalgae culture broth	99	HQ908763 *Labrenzia marina* strain F84034	99	*Alphaproteobacteria*
St11d-38	1	KX163508 basaltic subsurface ecosystems	99	KP866802 *Sphingomonas* sp. Sph16	99	*Alphaproteobacteria*
St11d-48	1	AB611565 hydrothermal environments	99	NR_044349 *Actibacter sediminis* strain JC2129	98	*Bacteroidetes*
St11d-3	1	KF886127 Prony Bay hydrothermal carbonate chimney	100	ND		*Bacteroidetes*
St11d-92	1	KM4110348 subsurface sulfidic cave stream	91	ND		*Chloroflexi*
St11d-47	1	EU283459 Anderson Lake	99	NR_044032 *Pelobacter seleniigenes*	96	*Deltaproteobacteria*
St11d-31	2	HM582730 halo-alkaline Lake Chitu shore sediment	99	NR_044032 *Pelobacter seleniigenes*	96	*Deltaproteobacteria*
ST11-6	1	KC574885 serpentinizing springs in The Cedars	94	KF148062 *Desulfurivibrio autodismutans*	93	*Deltaproteobacteria*
St11d-33	1	KJ149168 Prony Bay hydrothermal carbonate chimney	100	KF952448 *Desulfurivibrio alkaliphilus*	91	*Deltaproteobacteria*
ST11-3	1	AM778006 Cabeço de Vide Aquifer high pH groundwater associated to serpentinization	99	NR_109421 *Desulfosporosinus burensis*	91	*Firmicutes*
St11d-23	1	FJ638559 Terrestrial hydrocarbon seep in Taiwan	98	NR_043143 *Desulfonatronum cooperativum* strain Z-7999	97	*Firmicutes*
St11d-81	1	KJ149176 Prony Bay hydrothermal carbonate chimney	100	NR_115692 *Gracilibacter thermotolerans* JW/YJL-S1		*Firmicutes*
St11d-60	1	KJ149248 Prony Bay hydrothermal carbonate chimney	99	ND		*Firmicutes*
**St11d-73**	**10**	**KM207235 HPst091-1-1 this study**	**100**	**ND**		***Firmicutes***
St11d-17	1	KM978319 scorpion gut microbiota	99	HM801878 *Peptoniphilus* sp. 12-8	99	*Firmicutes*
St11d-56	2	HQ691962 stratified lagoon	99	NR_044010 *Thioalkalimicrobium microaerophilum*	99	*Gammaproteobacteria*
St11d-49	1	JF237196 skin, volar forearm	100	KT989843 *Moraxella osloensis*	99	*Gammaproteobacteria*
St11d-45	3	KJ475504 lab incubated oil slick from Gulf of Mexico oil spill	99	LC120333 *Legionella rubrilucens*	92	*Gammaproteobacteria*
St11d-70	4	KX022356 *Tyrophagus putrescentiae* - ham population	100	*Moraxella* sp. ALBL_051	100	*Gammaproteobacteria*
St11d-79	2	KF886081 Prony Bay hydrothermal carbonate chimney	99	ND		*Acetothermia*

3. Supplementary References

Altschul, S. F., Madden, T. L., Schäffer, A. A., Zhang, J., Zhang, Z., Miller, W., et al. (1997). Gapped BLAST and PSI-BLAST: a new generation of protein database search programs. *Nucleic Acids Res.* 25, 3389-3402.

Ben Aissa, F., Postec, A., Erauso, G., Payri, C., Pelletier, B., Hamdi, M., et al. (2014). Characterization of *Alkaliphilus hydrothermalis* sp. nov., a novel alkaliphilic anaerobic bacterium, isolated from a carbonaceous chimney of the Prony hydrothermal field, New Caledonia. *Extremophiles* 19, 183-188.

Bes, M., Merouch, M., Joseph, M., Quéméneur, M., Payri, C., Pelletier B., et al. (2015). *Acetoanaerobium pronyense* sp. nov., an anaerobic alkaliphilic bacterium isolated from the Prony alkaline Hydrothermal Field in New Caledonia. *Int. J. Syst. Evol. Microbiol.* 65(8), 2574-2580. doi: 10.1099/ijs.0.000307

Kelley, D. S., Karson, J. A., Blackman, D. K., Früh-Green, G. L., Butterfield, D. A., Lilley, M. D. et al. (2001). An off-axis hydrothermal vent field near the Mid-Atlantic Ridge at 30°N. *Nature* 412, 145-149.

Monnin, C., Chavagnac, V., Boulart, C., Ménez, B., Gérard, M., Gérard, E., et al. (2014). Fluid chemistry of the low temperature hyperalkaline hydrothermal system of Prony Bay (New Caledonia). *Biogeosciences* 11, 5697-5706.

Suzuki, S., Ishii, S., Wu, A., Cheung, A., Tenney, A., Wanger, G., et al. (2013). Microbial diversity in the Cedars, an ultrabasic, ultrareducing, and low salinity serpentinizing ecosystem. *Proc. Natl. Acad. Sci. USA* 110, 15336-15341.
